# Computers and the Internet: Tools for Youth Empowerment

**DOI:** 10.2196/jmir.7.5.e51

**Published:** 2005-10-04

**Authors:** Ruta K Valaitis

**Keywords:** Computers, Internet, adolescent, power, public health, schools, social change

## Abstract

**Background:**

Youth are often disenfranchised in their communities and may feel they have little voice. Since computers are an important aspect of youth culture, they may offer solutions to increasing youth participation in communities.

**Objective:**

This qualitative case study investigated the perceptions of 19 (predominantly female) inner-city school youth about their use of computers and the Internet in a school-based community development project.

**Methods:**

Youth working with public health nurses in a school-based community development project communicated with local community members using computer-mediated communication, surveyed peers online, built websites, searched for information online, and prepared project materials using computers and the Internet. Participant observation, semistructured interviews, analysis of online messages, and online- and paper-based surveys were used to gather data about youth’s and adults’ perceptions and use of the technologies. Constant comparison method and between-method triangulation were used in the analysis to satisfy the existence of themes.

**Results:**

Not all youth were interested in working with computers. Some electronic messages from adults were perceived to be critical, and writing to adults was intimidating for some youth. In addition, technical problems were experienced. Despite these barriers, most youth perceived that using computers and the Internet reduced their anxiety concerning communication with adults, increased their control when dealing with adults, raised their perception of their social status, increased participation within the community, supported reflective thought, increased efficiency, and improved their access to resources.

**Conclusions:**

Overall, youth perceived computers and the Internet to be empowering tools, and they should be encouraged to use such technology to support them in community initiatives.

## Introduction

The use of computers and the Internet can aid communities by supporting communication and access to information, thereby building social capital and community capacity [1,2]. Using computers can assist the community planning process [3], community participation, and information sharing [4-6]. Computer-mediated communication can build community awareness, encourage local decision making and dialogue between groups, and support disadvantaged communities. Status barriers can be reduced [7], and online communication with disenfranchised groups, such as women, people of color, and those with disabilities, can be promoted [8].

Youth are among the disenfranchised groups. Adults typically view youth as the cause of community deterioration rather than as a community asset [9,10]. Youth often feel they have little voice in their communities [11,12]. Youth participation in their communities can positively influence programs so that they are more responsive to youth’s needs [3] and can help support youth’s sense of self-determination from a community and individual perspective [4,5], thereby promoting their health. Increasing youth community participation, however, has been problematic. Since computers are an important aspect of youth culture, they may offer solutions to increasing and supporting community participation.

Youth have used computer technology to support various community projects [6-19]. Websites are seen as potential vehicles to support community building among groups that confront prejudice and domination [20]. Hart [21] argues that electronic publishing has the potential to broaden the reach of children’s voices and provide instant feedback. Cockburn [22] supports that children can use information and communication technologies to increase their participation in public life through better access to information, collective action, a more level political playing field, and the ability to include their views in decision making. Despite the claims of the benefits and barriers of computer technology, no research was found that addressed its use in community development work with youth.

The purpose of this qualitative case study was to explore youth’s use and perceptions of computers and the Internet as tools to support them in a school-based community development project. Objectives were to examine how youth used these tools and to explore youth’s perceptions of how the use of these tools affected participation within their community and communication with community members.

## Methods

### Study Design

This case study was conducted in a Southwestern Ontario inner-city school. Nurse facilitators (the school public health nurse, 2 senior nursing students, and the author) worked with 19 well-functioning grade seven and eight students for 12 weeks on a school-based community development project. Criteria for eligibility included demonstrated responsibility and the ability to manage academically by missing a 50-minute school period three times per week. The principal saw the community development project as an enrichment program for youth who could benefit from the challenge. To increase project sustainability, it was decided to include grade seven students who could carry on in the following school year. The principal selected a group of 35 students in grades seven and eight in consultation with teaching staff. Of the 35 who were selected, 23 consented to participate, and of these, 19 completed the project. Parental consent was also obtained. Participants were primarily female (79%, n = 15) and were evenly distributed between grade seven (n = 13) and eight (n = 12), including 6 participants who were visible minorities and 13 who were on the school honor roll. Many of the selected youth held other duties in the school, such as office and lunch hour supervision. A number of them came from single parent families. Initially, the youth generally felt that they had little voice in their community and were not trusted by adults [12].

Over 12 weeks, facilitators and youth met during school hours, three days per week, for one and a half hours each day. Youth identified needs and assets of their school and neighboring community, prioritized problems, and planned and implemented actions to improve their environment. Community assessment activities included mapping where youth live, work, learn and play; conducting a neighborhood walkabout; and photographing images to illustrate community needs and assets. The youth participated in a visioning session, interviewed key community members, conducted face-to-face interviews, and surveyed peers online about their views. Small groups of 4 to 8 youth worked on specific tasks with at least one adult facilitator. Large group sessions were also held to define community boundaries, decide on group goals, and share updates. Occasionally, a group of 7 youth met independently to refine computer and Internet skills and work on computer-based activities to support the project.

Participants identified many health issues, such as violence, drugs and alcohol, pollution, and smoking. They eventually chose to work on “do-able” school improvements. They enhanced the school’s general appearance by removing graffiti from school walls and beautified the school environment through a small greening project. They lobbied for improvements in washrooms and prepared two proposals: to establish a student-run school store and student council. Factors that enabled and constrained the community development process for youth are described elsewhere [23].

Youth accessed 6 computers in the library that were used to (1) construct websites containing their community assessment findings, (2) survey peers online, (3) create documents, (4) access information and resources through the Internet, and (5) communicate with each other, project facilitators, school staff, and community members using a computer-mediated communication system (FirstClass). This system, hosted by the local university, was used because it provided a private password-protected communication space for project participants. Participants could write private email messages or post messages to the group’s bulletin board, which could be viewed by all project participants. Community members, who were trained and given access to the system, could post and read all existing notes. This provided a useful archive of all messages. An example of one youth’s message enlisting help from an adult can be found in Textbox 1.

**Letter composed by a youth participant**

Hello. This is a message from the XXX School Community Development project. Hopefully, you [have received] this message [that was] sent to XXX Park. We have written this message to you in hopes that you will view our web site and give us ideas on how to improve our school with our “Greening and Cleaning” project to beautify our school. We could use any form of help from you. Maybe some people who work there or volunteer could visit XXX School and accompany us with some of the work. We are planning to start planting a small garden this spring. Maybe you could be able to help us plant and plan a long-term garden. Please e-mail us back, and if you can find any time please visit our web site at http://www.learnlink.mcmaster.ca/POWer.

Sincerely,

XXXX

### Research Methodology

This qualitative case study used multiple methods of data collection, including participant

observation, field notes, youth interviews (conducted individually, in pairs, or in a group of 3), facilitator interviews (conducted individually), online- and paper-based surveys, and quantitative analysis of computer-mediated communication notes. Interviews were transcribed, reviewed, and edited as needed. Table 1 outlines the timeline, method, and purpose of each data collection method. Ethics approval was obtained from the University of Toronto, Research Ethics Board.

**Table 1:** Data collection methods and purpose, in chronological order

**Time**	**Data Collection Method**	**Purpose**
Week 4	Open-ended, paper-based anonymous survey	To explore youth’s general feelings about the use of computers in the project
Week 9	Open-ended, online survey in the computer-mediated communication system	To explore youth’s perceptions about the community development project and computers
Weeks 1-13	Participant observation and raw field notes (Reflective notes were also added to the field notes.)	To obtain a detailed description of all project meetings, events, and communications with project participants (Reflective notes provided an audit trail.)
Weeks 14, 15	One-hour youth interviews (singly, in pairs, and, in one case, with a group of 3) (All interviews were taped, transcribed, checked, and edited for accuracy in transcription.)	To explore youth’s perceptions about the use of computers in the project
Weeks 14, 15	One-hour adult facilitator interviews (All interviews were taped, transcribed, checked, and edited for accuracy in transcription.)	To enhance the author’s interpretation, adult facilitators were interviewed about their impressions of youth’s perceptions of computers in the project.
End of study	Quantitative analysis of notes	To investigate youth’s involvement with online notes and to determine who wrote and opened notes

Richards and Richards suggest “working up from the data” and later reflecting and exploring it to form impressions and summaries [24] (p. 466). Qualitative coding of the interview transcripts, field notes, and open-ended surveys was conducted using this approach. The constant comparative method [25] was used to support the development of themes using qualitative analysis software (ATLAS.ti, ATLAS.ti Scientific Software Development GmbH, Germany). Two types of triangulation were used to build credibility of the qualitative findings [26]. One type used the constant comparison method, where themes were identified repeatedly, thus satisfying the existence of the theme. The second form was between-method triangulation. Interviews, participant observation, paper-based and online surveys as well as analysis of computer-mediated communication notes were used to gain an in-depth understanding of the phenomenon in question. Data can converge to a single proposition or demonstrate inconsistencies or contradictions. These differing outcomes in the analysis are valuable to consider to “construct meaningful propositions” or explanations about the data [27] (p. 15). The paper presents emergent themes, as well as inconsistencies uncovered in the analysis.

## Results

The first section briefly reports on technology use by youth. Following this, themes are presented related to youth’s perceptions. Quotes from male youth are marked as such.

### Use of Technology

Youth worked with computer technology in varying degrees: 18 (94.7%) youth developed Web pages, and 10 (52.6%) youth conducted an online survey of their peers’ opinions about community needs and assets. Of the 177 notes that were posted in the computer-mediated communication system, project youth posted 117 (66.1%) notes, whereas adults posted 60 (33.9%) notes. On average, youth created 6 notes (range 2-11, SD 2.4) and opened 20 notes (range 3-62, SD 15.7).

### Youth’s Perceptions of Technology

Four major themes describe youth’s perceptions of computer and Internet technology used in the project: reduced social risk factors, increased community participation, increased opportunity for reflection, and increased resources (Textbox 2). Inconsistencies are also presented.

**Themes and subthemes of youth’s perceptions of computer and Internet technology**

1. **Reduced Social Risk Factors**
  - Reduced anxiety
  - Increased control
  - Increased social status
2. **Increased Participation in Community**
  - Sharing youth’s views with the world
  - Getting others’ opinions
  - Getting access to influential people
3. **Increased Opportunity for Reflection**
4. **Increased Resources**
  - Increasing efficiency
  - Increased access to information and materials
  - Improved record of progress

#### Reduced Social Risk Factors

Three subthemes explain the major theme of reduced social risk factors. Youth felt that using computers reduced their anxiety, increased their control, and increased their social status.

##### Reduced Anxiety

Youth wrote to adults online to get information and advice. For example, youth emailed the local police liaison officer for ideas about vandalism prevention in the school and contacted the local botanical gardens for advice on plants suitable for a school greening project. Many youth felt that writing to adults via the Internet significantly lessened their anxiety compared to face-to-face or phone discussions. Youth were asked how they felt about constructing online messages to adults. SL (male) thought that “you wouldn't stutter or have trouble saying what you want.” This was explored further in interviews. PF explained, “Usually people get choked up over the [phone] line. If you write it, it is easier to say things.” Two girls described their experience:

LP [On the computer] it wasn't like when you’re in person. You’re kind of nervous with adults.

CB You don't want to say the wrong thing.

LP They get mad at you or something.

Computer-mediated communication was seen as neutral ground between youth and adults. YB (male) said, “If you talk face-to-face, it scares you.... On the Internet, it's okay. You don't know them, they don't know you. You [get] along.” Two youth who wrote to adult facilitators further explained the safe online environment:

CC On the computer you can say whatever you want. But when you are face-to-face you are afraid to tell [adults] what you want to do.... They might laugh at you. Laugh in your face. Tell you to get lost....

CP If you go up to any person and just start telling them you would probably feel shy....

CC The computer was easier. You can say whatever you want.

Online communication provided a safe way to initiate discussions with adults. CP explained how it was easier to write to the project facilitators at first. “We have to know the person before. We didn't know you guys and we didn't start talking to you guys at first.” As NT (male) explained, “[I’d rather] write [the police officer] an email first...because I never met the guy.” TS composed notes to reduce her anxiety before telephoning an agency. She preferred email because “I don’t have to talk.”

##### Increased Control

Some youth spoke about feeling more *in control* communicating with community members using computer-mediated communication. As described by SL (male), it “gives you more of a backbone.” Two youth appreciated being able to prepare their dialogue carefully for adults.

SL (male) [When talking] you sound kiddish. If you don't write anything like...

SG Big words.

SL (male) Yeah. I was ready to pull out a thesaurus and figure out what to say.

SG I think, umm, it was better on the computer than talking because...

SL (male) It's like, umm. Uhuhuh mmm. When you're talking.

SG Yeah exactly, they can't see you [online].

Youth felt more prepared to talk to adults because they could read their notes first. SL (male) stated, “It kind of gives you an idea of how they talk and how they think.” Writing on the computer also allowed youth to better prepare their notes. As MM noted, they could “spell check...and make sure the grammar is right.” Having time to read other’s notes and prepare their own gave youth a feeling of being more in control.

##### Increased Social Status

Communicating via the Internet raised youth’s perception of their social status. Many youth felt that they were more professional and were perceived as having “smartness” (YB, male). CB preferred communicating with adults online “because then people think you're smarter.” She later commented that writing to the principal online promoted being heard: “It was more professional.” A facilitator concurred that youth’s image was positively influenced through the use of technology: “I think they would have been seen differently by the community because they were using the technology.... [They] were seen as a bit smarter, brighter.” Youth and adults felt that youth’s credibility was also enhanced.

CP [On the computer] adults know we're not just jokers around that do whatever they want.

CC Yeah this isn't just kidding.

A facilitator reflected that “It certainly lent more credibility to the students' perception of what they were doing and external perceptions of what the students were doing within the school.” MM corroborated this view: “Sometimes they might not listen to you, because they hear your voice or something. They will actually read what you write.... They still know you're just a kid doing stuff. But they are more reading it, because you are not actually there looking at them. They are actually going to read it.”

Therefore using online communication raised youth’s perception of their social status and professionalism, in turn, increasing their credibility in the community.

##### Inconsistencies Regarding Increased Social Status

On two occasions, youth interpreted online messages from adults as negative and rude, leading to feelings of inferiority and low social status.

SG You're telling us to write proper and good and stuff, and then they come back and pretty much [call] us stupid.

SL (male) That's what I was thinking.

SG Yeah, thanks for your support. We're retards!

In one incident, a school police liaison officer replied to a youth’s online question, “What would you change in the community?” The officer replied, “Your first question is huge!! I need you to be more specific. I would change a lot of things if I had the authority to do so....” SL (male) interpreted this communication negatively: “[It] sounded like he was getting rude on the thing. But it was just the way he was talking. Sounded like he was saying...get smarter.” In another incident, US (male) wrote to the school staff member asking, “What kind of things do you think that this community needs?” The school staff member replied, “Is this a wish list?” CP spoke about her perception of this message:

CP He is giving you these good ideas. And then after he says, “Is this a wish list or something?” He should have put it down different. Not like that. When you think of a wish list you think like, “Oh, I want this, I want that.” Like you're being greedy.... You're not trying to be greedy. You are just trying to help out.

The school staff member reflected on the incident:

school staff member You have to choose your words carefully because words can be perceived differently. So in general, I'm quite careful with how I respond to email, and I read everything over before I send it, just to make sure the tone of it is the way I want it to be. But I think I was even a little more careful with the kids….

Youth were careful writing to adults. Although communicating online increased their sense of control, US (male) noted that, “It's different writing to the principal because I have to use proper grammar and stuff.... I was careful.” SL also felt “kind of nervous. [I held] back what I was going to [write]. Trying not to offend them.” A few youth felt awkward writing to adults. “I was used to writing to [my peers]. It was hard to write to someone older than I am” (BG). DC said, “I wasn't exactly worried, but it was hard [because] I couldn't exactly think of what to put down.” PF worried about the interpretation of her notes: “I don't know if they will like it, if they will understand it…if it shows the whole concept.” Thus, writing to adults was somewhat problematic for youth.

Although youth perceived themselves as smarter in the online environment and felt less anxiety overall, the process of online communication was not stress free. To protect their anonymity, cyber names were used, such as “Purrfection” and “Hellokitty.” This had the negative effect of reducing youth’s credibility in the eyes of community members. One community member was contacted by phone when an email response was not forthcoming; this community member considered the youth’s email a prank. To build credibility, youth began to describe the project in the body of their emails and referred to the project website once it was launched. This solved the problem.

#### Increased Community Participation

Youth felt that technology helped to increase their participation in the community. This is explained by three subthemes: sharing youth’s views with the community, getting other’s opinions, and getting access to influential people.

##### Sharing Youth’s Views With the Community

Youth felt strongly that they could share their thoughts about their neighborhood through websites. Willing youth were taught to construct a simple website containing ideas about their neighborhood and photographs. The Web pages reported on community problems, such as pollution, bullying, violence, and smoking. Areas of pride, such as their school, the local football stadium, and recreation center, were also highlighted. Project progress was also reported. In the interview, LP said, “We made websites so we could show people what we have done so far in the project.” NT (male) felt that the Internet helped youth share their ideas: “You can tell what we like and don't like about [our] communities.” MM felt that “more likely [youth] are going to write out what they think on the computer. They are not just going to come up and tell you.” Photographs augmented youth’s stories.

LP Because, you have pictures and stuff so kids could see. If there's just sentences and stuff it's like...

CB It's hard to imagine that in your head.

LP You might be thinking about something else but they're not talking about that. It might just sound like that.

CB Yes. I just think [the website] communicated it very well.

Web pages helped youth extend their reach into the community. CB explained, “People understood you better I guess.... If we didn't have the computers we wouldn't be able to reach the community that was the around us.” Unfortunately, youth’s websites were not well publicized. They were disappointed that only two school staff members reported visiting the site. Despite this, given another opportunity, all youth stated that they would build Web pages again. “It took us a lot of time [to build websites]. It's worth it” (YB). Youth highly valued the potential global reach of their ideas, even if it had no impact on the outcomes of the project itself. One facilitator felt this itself was empowering for youth:

facilitator [Working with computers] made them feel part of the bigger world. Whether or not they contributed to the community…they were certainly communicating with people out there, people that they might not have ever interviewed or connected with.... I think these kids were empowered in the sense that they knew that their work was displayed for the whole world to see, and I think that was empowering alone....

##### Getting Others’ Opinions

Youth gained peer input through the Internet, which helped them identify and prioritize community needs. Students in grades six to eight were asked to rate community problems and strengths in an anonymous online survey. Youth visited the classrooms and invited their peers to participate. LP wrote:

LP [Computers] helped us...figure out what we wanted to focus on. If we didn't have computers to do [online] surveys, we would probably take twice as long to figure it out. And we could chat with people to find out what they thought of what we have done.”

Two youth debated benefits and limitations of online surveys.

CP [I liked the online survey] because we got to get to results. We got a chance to use the survey to see the [opinions] of other people.

CC If we didn't have computers...we couldn't do as much as we did. Because we couldn't get other people's opinions unless they wrote us a letter or came face-to-face and talked to us.

CP I think it would be better with [face-to-face] interviews. You can get more answers. If you just give them a choice out of 3…they might have more to say. So on the computer, they can't write all their thoughts.

Despite the limitations of forced choice surveys, the majority of youth felt that computers helped them obtain other’s opinions about their community. MM stated, “We learned to take everyone into consideration no matter what they have to say. We learned that everything anyone says is a good idea, because it is [their] idea.” Clearly, youth valued their peer’s opinions and successfully included them in the project using technology.

##### Getting Access to Influential People

Some youth indicated that computers and the Internet provided access to influential people. DC wrote, “[I think computers have helped us] because we can ask important people if they have their own opinions on this community like [the police officer]....” BG shared that “[computers allowed] us to get important information from people outside of the school to help us with our project.” PF explained, “Without the computers, we would have never gotten replies and such from the [Royal Botanical Gardens] or people like that.” Through youth’s Web pages, communication notes, and the online survey, youth viewed computers and the Internet as effective means to communicate with community members, including influential adults, other youth, and the broader community.

#### Increased Opportunity for Reflection

As a result of the asynchronous nature of computer-mediated communication, writing thoughts online gave youth time to reflect. A few youth felt that this helped clarify others’ ideas. LP explained:

LP We could look and then we could come on another day and we could [write] to them about “What did you mean about that.” [Then we could answer] “I think that's a good idea” or something.

MM agreed:

MM It's going to be the same thing as you are going to say. But when you write it, you can think a lot more about what you are going to write. You don't have to worry about them being right there. You can go back and read it....

Facilitators observed that youth took their time to communicate to adults online. A facilitator shared in her interview that “[computers] gave [youth] a chance to generate responses that were more thoughtful.” CC noted that online polling provided more time for reflection compared to face-to-face interviews: “I think the computer is easier because people get to think more. If you're face to face…like if you're thinking a long time, people get might get bored of me.” Technology provided more opportunity to think before acting.

#### Increased Resources

Participants felt that computers increased resources. This is explained by three subthemes: increased efficiency, increased access to informational and material supports, and a permanent record of progress.

##### Increased Efficiency

Computers and the Internet increased youth’s efficiency in accessing resources. SL (male) felt that “you can get information faster and cheaper.” CP explained, “The computer [online survey] gave you faster results.” MM felt that technology provided a reliable and efficient means to communicate:

MM [By using computers] you know it's going to get to them. If you just call and leave a message, you have to worry about them calling you back. You can just check your email to see if they emailed you back. No waiting for them to call you back at a certain time.

Increased efficiency was also noted in a facilitator’s interview:

facilitator Email is quick and easy and accessible to everyone. At least that's the perception I think most of these students had.... [Youth] were able to write emails as soon as we found the sites that intrigued us or came up with ideas, and it was a very quick way, again, to start that dialogue…. They were able to do things right away.

Youth, therefore, perceived a heightened sense of immediacy and access to quick feedback.

##### Information and Material Resources

Participants felt that the Internet enhanced the group’s ability to search for and access community agencies, information, and material resources. Some youth searched for community contacts, such as the police and parks and recreation staff. They gathered information about forming a school council and starting a school greening project. MM felt that “[the Internet] helped with all our information that we needed to explore.” Youth successfully obtained material resources. LP stated, “[through email we could] write to each other and to people [that] we needed supplies from.” GC wrote, “I think the computers have helped to get donations from other people.” PF wrote, “If we didn't have these computers, we would have never been able to email all those people that could help us.” The project website helped publicize their project goals, which resulted in access to free resources. For example, the Children’s Museum staff viewed the project website and then waived a room booking fee for a group function.

##### Permanent Record of Progress

Some youth felt that computer-mediated communication was valuable to document and store information. SL (male) identified that “the program holds information, [and] you can lose paper.” Later, he commented online that “[computers] have helped us a lot by storing information….” MM indicated that “we wrote a lot of our ideas on the computer, and with that we can come back and read those ideas any time we want.... We will never have to worry about forgetting them.” Thus, computer-mediated communication maintained a permanent record of project communications.

### Inconsistencies Regarding Computers and the Internet as a Resource

A small number of participants generally disliked technology. They felt that using technology was time-consuming. BM felt “the logging in would take forever. And then you had all your mail you had to check. And then you had to do what you had to do, and it would take forever.” GG spoke about contacting the local park: “[I would prefer] to phone because it will get there quicker.” SM, who was very artistic, shared the following in the interview:

SM I don't like computers. If I would just [have] written it, it would have taken less time.... And it takes a long time to get into the program and we had to cut and paste it and all that stuff. I could have just written it and put it on pretty paper.

Computers were not appealing to all youth. Many justifiably complained about slow connections and the Internet frequently being down.

## Discussion

The major themes and subthemes that have been presented closely parallel a number of factors associated with powerlessness and empowerment in Wallerstein’s Empowerment Education Model [28]. Therefore, these themes are interpreted using this model. Textbox 3 lists factors from the model that appear congruent with the major themes. The model supports the finding that youth perceived computers and the Internet to (1) reduce certain social risk factors thought to be associated with powerlessness and (2) to increase factors that are associated with empowerment. Despite technology limitations, computers and the Internet generally appear to be empowering tools for youth.

**Major concepts from Wallerstein’s empowerment education model [28] that parallel themes**

- **Powerlessness**
  - Social Risk Factors
  - High psychological demands
  - Low control
  - Low in hierarchy
- **Empowerment**
  - Increased Participation in Decision making
  - Critical Thinking/Conscientization
  - Resource Equity/Access

### Overcoming Threats to Empowerment with Computers and the Internet

The themes—reducing levels of anxiety, gaining control, and increased social status—were related to online communication with adults. These themes parallel social risk factors in Wallerstein’s model [28], namely high psychological demands, a feeling of low control, and being low in social hierarchy. Reduction of these factors supports empowerment. Computers and the Internet can, therefore, be viewed as supportive tools for youth to overcome threats to empowerment. Youth felt more confident, better prepared, and more knowledgeable about the adults with whom they communicated online.

Others found similar benefits from working with technology. Resnick et al [19] worked with youth in computer clubhouse projects where design experiences supported learning by giving youth a sense of control over the learning process. In this case study, youth felt in control communicating to adults online since they could manipulate the medium. They also felt increased status. Similarly, in an online nurse practitioner program, students identified that the novelty of taking a computer-based course and greater computer knowledge raised their status among their colleagues and families [29]. Reductions of social status differences have also been noted in computer-mediated communication research with adults in their work environments [7]. Further research into the impact of technology on communication processes between adults and youth is warranted.

### Supporting Empowerment With Computers and the Internet

Wallerstein places emphasis on increasing participation in decision making to support empowerment [28]. Youth felt an increased sense of participation with their neighborhood and school community through online communication. They also gained others’ perspectives using computer-mediated communication and online polling and, more importantly, shared their personal views on Web pages and in communication notes. Youth considered their website to be the most valuable strategy to express their ideas to the broader community. Resnick et al indicated that designing computer projects “facilitated personal connections to knowledge, because designers often develop a special sense of ownership (and caring) for the products (and ideas) that they design” [19] (p. 270). Perhaps this explains, in part, the value that youth placed on this activity. Wong et al [30] indicated that designing Web pages was a popular activity in Michigan elementary school computer clubs. They attributed this to three factors: providing youth with an authentic learning experience, increased participation with the broader community, and an increased sense of achievement. Community practitioners are thus encouraged to support youth in website creation.

This study supports websites as an effective narrative tool for youth. Rees [31] sees benefits from youth telling their story, in which a form of narrative therapy occurs and mutual education and sharing between young people and adults begins. Similarly, Schwab [32] reviewed community development initiatives and found that when youth tell their story, they grow. Media tools such as journals, masks, and drama that represent youth’s lives were identified as valuable tools for self-expression and advocacy. Other creative methods include murals [33], zines (self-published magazines) [34], and videos [35]. Other researchers [36-39] describe using photography as a voice for community issues and as a method for participatory research and community assessment.

Most youth reported showing their personal Web pages to family and friends at home or at the public library. Despite the limitations for those without home Internet access, the potential for broad community reach through the websites and email was empowering. Wallerstein [28] indicated that stronger social networks support community empowerment. Networks can be enhanced through websites and online communication. Newer technologies such as Web conferencing and instant messaging have this potential.

Computers and the Internet were viewed by most youth as valuable resources. Wallerstein refers to a lack of resources, such as finances or access to systems, as a risk factor related to powerlessness, whereas access to resources supports community empowerment [28]. CB reflected that “I have learned that you can change your community if you have a lot of resources and a lot of people to help.” Most youth felt that computers increased their efficiency; gave them more control; made information, people, and materials more accessible; and were useful for storing their ideas. Some youth commented that the time afforded to think and construct responses on the computer was beneficial. Bamberger [40] described work with youth in student labs at the Massachusetts Institute of Technology, which provided an environment for youth to “catch up with their own understandings—slowing down events and actions so as literally to grasp the ‘goings on’ of things and how they relate to ideas” (p. 239).

### Limitations of Computers and the Internet

Youth noted limitations in using technology that had the potential to increase their feelings of powerlessness. Although online communication generally increased youth’s perception of social status, at times, youth interpreted adults’ online comments as “put downs.” Community practitioners are cautioned when working with youth to word their email messages carefully. Care needs to be taken in selecting cyber names for youth. Since youth participants selected creative cyber names (eg, Sir Lancelot), their credibility was weakened. Further research into the impact of adults’ different writing styles on youth’s perceptions, and vice versa, is needed. Although synchronous communication was not used, one youth suggested “chatting” with neighboring schools. There is significant potential for synchronous communication to support community networks. A Canadian study by Skinner et al [41] found that the quality of Internet access for health information and resources was affected by privacy, gate-keeping, timeliness, and functionality. It would seem reasonable to consider these factors when applying computers and the Internet as tools to support future youth-driven community development initiatives.

A few youth indicated disinterest in computers and rarely chose to work with them other than to build Web pages; they were also indifferent or negative about the impact of computer technology on the project. Further research into what limits youth’s engagement with computers, how computers can be used to serve more authentic purposes in community development, and how they can affect youth’s sense of empowerment is needed.

### Conclusions

Overall, youth perceived computers and the Internet to be empowering tools in this community development project. Youth felt better able to participate in the community. The Internet provided a safe way to communicate with the neighborhood and school community by supporting youth’s ability to obtain others’ views and share their own. By communicating online, youth perceived themselves to have higher social status and increased credibility. The Internet’s potential to reach the broader community was empowering in itself. Technology was viewed as a useful resource that improved youth’s efficiency, supported critical reflection, and created a permanent record of their work. Despite the benefits, technical problems, computer access issues, the potential for online miscommunication, and the potential to raise youth’s feelings of inadequacy were drawbacks of using technology. This case study involved mostly female students who were high academic achievers. Thus, transferability of these results is limited to similar populations. It is unknown how results might differ with students with lower academic achievement or with a different gender mix.
